# The Evaluation of the Body Weight Lowering Effects of Herbal Extract THI on Exercising Healthy Overweight Humans: A Randomized Double-Blind, Placebo-Controlled Trial

**DOI:** 10.1155/2013/758273

**Published:** 2013-10-29

**Authors:** Soo Hyun Cho, Yoosik Yoon, Young Yang

**Affiliations:** ^1^Department of Family Medicine, College of Medicine, Chung-Ang University Hospital, Seoul 156-755, Republic of Korea; ^2^Department of Microbiology, College of Medicine, Chung-Ang University, Seoul 156-756, Republic of Korea; ^3^Department of Life Science, Sookmyung Women's University, Seoul 140-742, Republic of Korea

## Abstract

We investigated the effects of herbal extracts, a mixture of Scutellariae Radix and Platycodi Radix containing the active ingredients Baicalin and Saponin (target herbal ingredient (THI)), on lowering body weight. The present study was a prospective, randomized, double-blind, and placebo-controlled trial carried out at the outpatient department of a hospital over a period of 2 months. Group 1 patients (*n* = 30) received THI, and group 2 patients (*n* = 23) received placebo three times a day before meals. Weight, waist circumference, BMI, total cholesterol, triglycerides, HDL cholesterol, LDL cholesterol, and glucose were measured at baseline and again at the 2nd month. For safety evaluation, various hematological and biochemical parameters were assessed. Values of mean change of weight in the THI-treated group were −1.16 ± 1.41 kg and in the placebo-treated group were −0.24 ± 1.70 kg, respectively. The difference in mean change of weight in the THI-treated group compared with that in the placebo-treated group was statistically significant (*P* < 0.05). The incidence of subjective and objective adverse drug reactions was insignificant (*P* > 0.05). THI was statistically significant in its effectiveness on the weight loss.

## 1. Introduction

Overweight or obesity substantially raises a patient's risk of morbidity from hypertension [1, 2], type 2 diabetes [3, 4], dyslipidemia [5], cardiovascular disease (CVD) [5], stroke [6], gallbladder disease [7], osteoarthritis [8], sleep apnea, respiratory problems [9, 10], and also endometrial, breast, prostate, and colon cancers [11]. Overweight and obesity are a major public health concern not only in western countries but also in Asian countries because of its increasing prevalence and its association to morbidity and mortality [12, 13]. The prevalence of obesity (body mass index (BMI) ≥ 30 kg/m^2^) in USA was 32.2% in 2004 [14]. While the prevalence of obesity in Asian populations is lower than that of Caucasians, the health risks associated with obesity occur at a lower BMI. Accordingly, the criteria for overweight and obesity in the Asian-Pacific region of WHO has been proposed as BMI ≥ 23 kg/m^2^ and BMI ≥ 25 kg/m^2^, respectively [15]. The prevalence of obesity using a BMI ≥ 25 kg/m^2^ among Koreans over age 19 was 30.8% in 2010 [16] which represented a rapid increase in comparison to rates in 1998 [17].

There is strong evidence that weight loss in overweight and obese individuals reduces risk factors for diabetes and cardiovascular disease. Strong evidence exists that weight loss reduces blood pressure in both overweight hypertensive and nonhypertensive individuals, reduces serum triglycerides, increases high-density lipoprotein (HDL) cholesterol, and generally produces some reduction in total serum cholesterol and low-density lipoprotein (LDL) cholesterol. Weight loss reduces blood glucose levels in overweight and obese persons without diabetes; weight loss also reduces blood glucose levels and HbA1c in some patients with type 2 diabetes. Although there have been no prospective trials to show changes in mortality with weight loss in obese patients, reductions in risk factors would suggest that development of type 2 diabetes and CVD would be reduced with weight loss [18]. Therefore, drugs or supplements to help weight loss need to be developed.

This study is aimed at investigating the body weight lowering effect of extracts from a mixture of herbs with the active ingredients Baicalin and Saponin (target herbal ingredient (THI)). Baicalin is the active ingredient in Scutellariae Radix (the root of *Scutellariae baicalensis*) which has been shown to be efficacious in reducing weight, visceral fat mass, serum cholesterol, free fatty acid, and insulin concentrations on high-fat diet-fed rats in comparison to controls [19]. High-fat diet-fed rats were also shown to suppress expected increases in body weight [20, 21], plasma triacylglycerols, adipose tissues [20, 22], food consumption, expression of leptin, and neuropeptide Y [21] and prevented hepatic steatosis [22] after consuming saponin isolated from ginseng or Platycodi Radix. The present study was a prospective, randomized, double-blind, and placebo-controlled trial carried out at an outpatient department of the hospital over a period of 2-months.

## 2. Materials and Methods

### 2.1. Study Subjects

Subjects who participated in the study were all healthy volunteers who were recruited using announcements in leaflets and posters at Chung-Ang University Hospital, Seoul, Korea. The study was approved by the Institutional Review Board (IRB) of the Chung-Ang University Hospital (Ethics Committee reference number C2010114 (410)). In addition, written informed consent was obtained from all subjects before initiation of the trial.

From November 2010 to December 2010, a total of 80 patients visited the Outpatient Department of Family Medicine of Chung-Ang University Hospital where they had a baseline clinical examination (weight, BMI, body and composition) and laboratory investigations. Body composition was assessed by a bioelectrical impedance analysis system (InBody 720, Biospace Co., Seoul, Korea) that was developed for the Korean population.

### 2.2. Inclusion and Exclusion Criteria

The inclusion criteria were patients with BMI ≥ 23 kg/m^2^ of either sex between the age of 18 and 70 years. The following categories of patients were excluded: patients with endocrinologic obesity; patients using other medications that can alter body weight or lipid levels; patients with clinically significant cardiovascular, respiratory, or hepatobiliary disorder; chronic diarrhea; and pregnant or lactating women.

### 2.3. Preparation of Herbal Dietary Supplement 

The dietary supplement, THI, was a liquid herbal extract of Scutellariae Radix and Platycodi Radix as its main ingredient. The two herbs were extracted and made into a dietary supplement by Namil Farm & Ginseng Co. (Geumsam, Korea). The two herbs were mixed at a 1 : 1 ratio and extracted with 70% ethanol. The ethanol was then evaporated under low pressure conditions. The final yield of the herbal extract to raw herbs was 25% (w/w). For the standardization of the herbal extract, the contents of Baicalin—an ingredient of Scutellariae Radix—and Saponin—an ingredient of Platycodi Radix—were measured using high performance liquid chromatography resulting in 5.16% of Baicalin and 3.95% of Saponin (w/w). The preclinical study of this herbal extract was reported previously [22]. Each pouch of THI supplement contained 2.28 gm of herbal extract as its main ingredient, 3.18 gm of oligosaccharide as a sweetener, and 0.05 gm of berry flavor and was adjusted to the final volume of 50 mL with water. Placebos, with no herbal extract, contained same sweeter and flavor to have a similar appearance and scent.

### 2.4. Sample Size

There were no previous studies on the efficacy of THI with respect to body weight lowering effects for healthy overweight humans. We postulated that we would need a sample size of *n* ≥ 36 in order to see standard deviation and difference of body weight change between groups with a statistical significance level of 0.05 and power of test of 0.9 based on previous animal study [23]. Therefore it was decided to enroll 60 subjects in the study anticipating a 40% exclusion from the study.

Initially, 69 subjects were enrolled in the study. Fifteen subjects were either disqualified for not complying with the drug regimen or voluntarily dropped from the study. One subject was excluded for excessive exercise. A final sample size of *n* = 53 was used in the final analysis and reported in the results.

### 2.5. Methods and Statistics

A total of 69 patients were enrolled in the study according to inclusion and exclusion criteria and were randomized into two groups of 39 patients for THI and 30 patients for placebo on the computer-generated list by SAS randomization program at Chung-Ang University, College of Medicine, Department of Microbiology. Group 1 patients received THI three times a day before meals. Group 2 patients received placebo three times a day before meals. After enrollment into the study, tests were carried out for baseline clinical examination (weight, BMI, body composition (muscle (kg), fat (kg), and fat ratio (%)), and blood pressure) and laboratory investigations; hematological tests (red blood cell count, hemoglobin level, (Hb), hematocrit level, white blood cell count, differential white blood cell count, and blood platelet count); blood biochemical tests (total protein, albumin, total cholesterol, HDL cholesterol, LDL cholesterol, triglycerides, blood urea nitrogen, uric acid, serum creatinine, aspartate transaminase (SGOT), alanine transaminase (SGPT), *γ*-glutamyl transpeptidase (*γ*GTP), and blood glucose); urine analysis (urine protein, urine sugar, and urinary sediment (erythrocytes, leukocytes, and casts)); and Serologic tests (highly sensitive C-reactive protein (hs-CRP), thyroid stimulating hormone (TSH), and free T4)). In the same visit, irrespective of group allotted, all the patients were educated about the importance of lifestyle changes including healthy dietary habits and exercise in weight reduction and maintenance. Patients were given information about the nutritional value of various foods and few simple exercises for decreasing and maintaining near-normal body weight by a clinical dietician. Their compliance for lifestyle change advice was checked through verbal questions at each visit. Biochemical examinations assessed were above laboratory investigations (hematological tests, lipid profile, liver function test, blood glucose, serum creatinine, urine analysis, etc.) at baseline, the 1st month, and the 2nd month of study period. The efficacy of the drug was assessed by primary efficacy parameters, that is, body weight (kg), BMI (kg/m^2^), waist circumference (in cm measured at midpoint between the lower border of rib cage and iliac crest), and body composition. Other parameters assessed were total cholesterol (mg/dL), triglyceride (mg/dL), LDL cholesterol (mg/dL), HDL cholesterol (mg/dL), random blood glucose level (mg/dL), hs-CRP (mg/L), and blood pressure (mmHg). Safety was assessed in terms of both subjective and objective adverse effects with grading scale of Common Terminology Criteria for Adverse Events v3.0 (CTCAE) [24]. Subjective symptoms such as anorexia, nausea, indigestion, loose stools, and constipation were assessed by questioning patients at each visit. Objective signs were assessed by clinical and biochemical examination. Followups were performed at the 1st and 2nd months of the study period, and during each visit, all the efficacy parameters were measured, and safety evaluations were done.

Quantitative data was analyzed by *t*-test or Mann-Whitney *U*-test for the differences between two means, while qualitative data was analyzed by Chi-square test or Fisher's exact test for difference between two proportions. *P* value < 0.05 was taken as significant, while *P* value > 0.05 was taken as insignificant.

Analyses were performed using the SPSS statistical software Version 12.0KO for windows (SPSS Inc.).

## 3. Results

Nine patients withdrew from trial in the THI-treated group, and 6 patients withdrew from trial in the placebo-treated group because they were busy and had been taking their supplements irregularly. No patients withdrew due to adverse effects of the supplements. In the placebo-treated group, one male patient was excluded because, unlike the rest of the patients, he exercised over 2 hours a day which was too vigorous for the purpose of this study.

At the time of the final analysis, there were 30 patients in the THI-treated group and 23 patients in the placebo-treated group.

Baseline values of both groups were comparable with respect to age, sex, weight, height, BMI, waist circumference, hip circumference, blood pressure, and laboratory investigations (random blood glucose level, total cholesterol, triglyceride, HDL cholesterol, LDL cholesterol, etc.) (Table 1). There were insignificant differences between the two groups except the total bilirubin level (*P* < 0.05).

In the THI-treated group, the mean weight at baseline was 71.84 ± 10.57 kg and reduced to 70.68 ± 10.25 kg 2-months later. In the placebo-treated group, mean weight at baseline was 67.89 ± 7.85 kg and reduced to 67.64 ± 7.96 kg after 2-months.

When the mean value of the change of weight in the THI-treated group was compared with that in the placebo-treated group, it was found that the difference in mean change of weight was statistically significant (*P* < 0.05) (Table 2). When values of the mean change of the other clinical examination variables in the THI-treated group were compared with that in the placebo-treated group, the differences were found to be statistically insignificant (*P* > 0.05).

There were no significant (*P* > 0.05) differences in the mean change of blood glucose level, total cholesterol concentration, triglyceride concentration, HDL cholesterol concentration, LDL cholesterol concentration, hs-CRP level, and systolic and diastolic blood pressure during the 2-month treatment in the THI-treated group compared to the placebo-treated group (Table 2). All changed laboratory investigations were in normal range.

In the present study, the incidence of adverse drug reactions like nausea (16.7%), indigestion (26.7%), abdominal pain (3.3%), loose stools (6.7%), diarrhea (13.3%), constipation (6.7%), insomnia (6.7%), itching (6.7%), satiety (6.7%), suppression of appetite (10.0%), and so forth was insignificant in the THI-treated group compared to the placebo-treated group (*P* > 0.05) (Table 3). Most of these adverse effects were mild and transient. THI had no adverse impact on white blood cell count, hemoglobin, red blood cell count, hematocrit, platelet count, total protein, albumin, total bilirubin, direct bilirubin, SGOT, SGPT, *γ*GTP, blood urea nitrogen, serum creatinine, uric acid, TSH, and free T4 level at the end of study (Table 2).

## 4. Discussion

A sedentary lifestyle coupled with an increased intake of energy-dense food is contributing to the increased prevalence of obesity worldwide. A range of strategies can be employed for weight loss which includes lifestyle changes (diet, exercise), behavioral therapy, pharmacotherapy, and surgery. Orlistat is an effective and well-tolerated antiobesity drug, which can be employed as an adjunct to therapeutic lifestyle changes to achieve and maintain optimal weight [25]. But most antiobesity drugs act on the central nervous system to suppress appetite, reduce food intake, and have serious adverse effects. Therefore, alternative drugs or supplements to help weight loss need to be developed.

In traditional medicine of East Asian countries including China, Korea, and Japan, Scutellariae Radix extracts are widely used for clinical treatment of hyperlipemia, atherosclerosis, hypertension, dysentery, the common cold, and inflammatory diseases such as atopic dermatitis. Baicalin, 5, 6-dihydroxyflavone-7-glucuronic acid, is a major flavonoid found in Scutellariae Radix and is well known for its anti-inflammatory and antioxidant activities [26, 27], and few reports describe the antiobesity effects of this compound. In vitro study, Baicalin treatment of 3T3-L1 preadipocytes was shown to inhibit triglyceride accumulation and lipid droplet formation during induced adipogenesis. Baicalin inhibits adipogenesis through the downregulation of proadipogenic genes, including PPAR*γ*, C/EBP*α*, and KLF15, as well as the upregulation of antiadipogenic regulators, including C/EBP*γ* and KLF2 [28]. Also in animal studies, Baicalin might have beneficial effects on the development of hepatic steatosis and obesity-related disorders by targeting the hepatic AMPK [19].

Platycodi Radix has long been used as an expectorant in traditional oriental medicine. Recently, it was reported that Platycodi Radix has anti-inflammatory, antiallergy, antitumor, apoptosis-inducing, and immune-stimulating activities [29–31]. Recently, it was reported that platycodin D, a major saponin component of Platycodi Radix, inhibits fat accumulation and adipogenesis [22, 32–35]. The antiobesity effect of the crude saponins in mice fed a high-fat diet may be due to the inhibition of intestinal absorption of dietary fat by platycodin D [22]. And the antiadipogenic effect of platycodin D involves the upregulation of KLF2 and subsequent downregulation of PPAR*γ* [36].

In our study, values of mean change of weight during the 2-month treatment in the THI-treated group were −1.16 ± 1.41 kg and in the placebo-treated group were −0.24 ± 1.70 kg, respectively. When the mean change in weight in the THI-treated group was compared with that in the placebo-treated group, it was found that the difference in the mean change of weight was statistically significant (*P* < 0.05), but the mean change of weight during the 2-month treatment in the THI-treated group was −1.16 ± 1.41 kg. Caloric restriction combined with exercise or alone to create a daily energy deficit is the foundation of most weight loss programs. In these programs, many individuals successfully lose at least 4.5 Kg of body weight through behavioral approaches for 6 months [36]. In other studies, the placebo-treated group loses at least −1.49 ± 6.19 kg of body weight during the 2-months [25]. So can we conclude from our study that THI was effective in weight loss?

Our study was conducted in South Korea between November of 2010 to February of 2011, when most Koreans eat and drink more than usual. In addition, the winters in Korea frequently have heavy snowstorms, so our patients did not exercise properly around the time we were completing our study. In our study, even though weight loss was small, THI was statistically significant in its effectiveness on the weight loss.

Prior to this study, THI improved metabolic abnormality in mice that were fed high-fat diets [23]. And the extracts of Platycodi Radix with white balloon flower ameliorated obesity and insulin resistance in obese mice via the activation of AMPK/ACC pathways and reductions of adipocyte differentiation. It also reduced the elevated circulating mediators, including triglyceride, total cholesterol, leptin, resistin, and monocyte chemotactic protein- (MCP-) 1 in obesity [37].

Therefor we expected that THI improved metabolic abnormality with body weight loss in our study. In our study, the values of mean change of triglyceride and HDL cholesterol during the 2-month treatment in the THI-treated group seemed to be improved than those in the placebo-treated group. However, those were not statistically significant (*P* > 0.05), because the remedy was a supplement, not a medication, the group was small, the study period was short, and the body reduction effect was too small. We will, therefore, need further research in order to make conclusions about its long-term effects.

However, in our study, values of mean change of platelet count during the 2-month treatment in the THI-treated group seemed to be decreased than that in the placebo-treated group. But, that was not statistically significant (*P* > 0.05). Scuttellaria has antithrombotic and antiplatelet actions that prevent the pathological changes of atherosclerosis and restenosis because it has several chemical compounds. Baicalin has antiproliferative and lipoxygenase-inhibitory activities [38], and Oroxylin A has antithrombotic activities [39]. Also there was a protective effect from *Scutellaria baicalensis* Georgi on cerebral ischemia injury [40]. So if we studied for a longer time, the result would be statistically significant, and the side effect of bleeding tendency would occur. But in our study the change occurred in normal range.

The adverse drug reactions were indigestion (26.7%), nausea (16.7%), diarrhea (13.3%), and loose stool (6.7%). They were mainly gastrointestinal symptoms. These adverse drug reactions may have been due to the body weight lowering effects of the crude saponins, the inhibition of pancreatic lipase activity and intestinal absorption of dietary fat by platycodin D [28]. However, patients who suffered from constipation before the study began felt that the supplement relieved their constipation (16.7%). In our study, the incidence of subjective and objective adverse drug reactions was insignificant in the THI-treated group compared to the placebo-treated group (*P* > 0.05).

We could not make a capsule or tablet for this study because the THI supplements were 50 mL of liquid extracts. There can, therefore, be problems for long-term storage.

## 5. Conclusion

In conclusion, the herb extract, a mixture of Scutellariae Radix and Platycodi Radix containing the active ingredients Baicalin and Saponin (target herbal ingredient (THI)) was statistically significant in its effectiveness on the weight loss without significant side effects, which was demonstrated by our present randomized, double-blind, and placebo-controlled clinical trial.

## Figures and Tables

**Table 1:** General characteristics of study subjects.

Variables	THI	Placebo	*P* value
	(*n* = 30)	(*n* = 23)	
Sex			
Male	3 (10)	5 (21.7)	0.272
Female	27 (90)	18 (78.3)	
Age (years)	42.90 ± 12.67	41.83 ± 14.82	0.778
Height (cm)*	159.22 ± 7.91	160.03 ± 9.46	0.654
Weight (kg)	71.84 ± 10.57	67.89 ± 7.85	0.139
Body mass index (kg/m^2^)	28.35 ± 3.95	26.51 ± 2.21	0.050
Waist circumference (cm)	84.02 ± 8.66	82.96 ± 6.61	0.628
Hip circumference (cm)*	102.79 ± 7.09	99.40 ± 3.97	0.337
Waist/hip (%)	81.75 ± 6.51	83.50 ± 6.28	0.329
Body composition			
Muscle (kg)*	42.60 ± 7.91	41.14 ± 8.07	0.355
Fat (kg)	26.68 ± 7.94	24.43 ± 4.24	0.225
Fat ratio (%)	36.90 ± 7.81	36.28 ± 6.48	0.759
Blood pressure (mmHg)			
Systolic	124.80 ± 13.66	125.17 ± 13.58	0.922
Diastolic*	85.47 ± 10.18	84.74 ± 9.79	0.842
Blood glucose (mg/dL)*	94.73 ± 11.43	96.00 ± 6.19	0.570
Total cholesterol (mg/dL)	197.20 ± 38.39	201.39 ± 39.83	0.700
Triglyceride (mg/dL)*	115.03 ± 76.11	117.57 ± 56.93	0.341
HDL cholesterol (mg/dL)*	49.97 ± 9.04	54.04 ± 12.55	0.261
LDL cholesterol (mg/dL)	117.67 ± 31.76	115.91 ± 28.30	0.836
hs-CRP (mg/L)*	0.92 ± 0.88	2.41 ± 6.52	0.495
White blood cell count (10^*∧*^9/L)*	5.99 ± 1.37	6.72 ± 1.56	0.097
Hemoglobin (g/dL)	13.69 ± 1.23	14.11 ± 1.42	0.262
Hematocrit (%)*	41.03 ± 3.25	42.22 ± 3.65	0.274
Platelet count (10^*∧*^9/L)*	256.97 ± 43.91	255.87 ± 45.20	0.641
Total protein (g/dL)	7.26 ± 0.25	7.42 ± 0.42	0.091
Albumin (g/dL)*	4.32 ± 0.25	4.36 ± 0.25	0.562
Total bilirubin (mg/dL)*	0.58 ± 0.16	0.74 ± 0.29	0.029^‡^
Direct bilirubin (mg/dL)*	0.17 ± 0.05	0.21 ± 0.08	0.091
SGOT (IU/L)*	23.10 ± 7.22	21.30 ± 5.49	0.335
SGPT (IU/L)*	25.77 ± 14.86	22.17 ± 13.00	0.243
*γ*GTP (IU/L)*	32.53 ± 29.19	24.65 ± 15.08	0.596
Blood urea nitrogen (mg/dL)*	13.33 ± 3.16	13.70 ± 3.75	0.704
Serum creatinine (mg/dL)*	0.79 ± 0.16	0.83 ± 0.12	0.332
Uric acid (mg/dL)*	4.31 ± 1.03	4.57 ± 1.49	0.713
TSH (mIU/mL)*	2.23 ± 1.58	1.95 ± 1.19	0.634
Free T4 (ng/dL)*	1.08 ± 0.19	1.10 ± 0.14	0.389

Chi-square test (categorical variables); *t*-test (continuous variables); *P value using Mann-Whitney *U*-test; ^‡^ *P* < 0.05.

Values given are number of patients (%) or mean ± SD.

LDL: low-density lipoprotein; HDL: high-density lipoprotein; hs-CRP: highly sensitive C-reactive protein; SGOT: aspartate transaminase; SGPT: alanine transaminase; *γ*GTP: *γ*-glutamyltranspeptidase; TSH: thyroid stimulating hormone.

**Table 2:** Changes over time in clinical examination and laboratory investigations between THI and placebo ingestion groups.

Variables	THI	Placebo	*P* value
	(*n* = 30)	(*n* = 23)	
Weight (kg)	−1.16 ± 1.41	−0.24 ± 1.70	0.036^‡^
Body mass index (kg/m^2^)	−0.41 ± 0.55	−0.10 ± 0.70	0.083
Waist circumference (cm)*	−1.77 ± 1.91	−1.04 ± 2.38	0.156
Hip circumference (cm)	−1.23 ± 1.77	−0.71 ± 1.78	0.304
Waist/hip (%)	−0.77 ± 2.23	−0.46 ± 2.32	0.628
Body composition			
Muscle (kg)*	−0.63 ± 1.51	−0.06 ± 0.98	0.151
Fat (kg)	−0.50 ± 1.36	−0.38 ± 1.47	0.761
Fat ratio (%)*	−0.06 ± 1.82	−0.41 ± 1.36	0.957
Blood pressure (mmHg)			
Systolic*	−11.30 ± 13.92	−5.65 ± 16.29	0.368
Diastolic	−8.20 ± 10.60	−6.00 ± 11.07	0.466
Blood glucose (mg/dL)*	−1.93 ± 8.05	1.43 ± 7.26	0.062
Total cholesterol (mg/dL)	−4.63 ± 26.84	−9.73 ± 24.23	0.478
Triglyceride (mg/dL)*	−14.96 ± 59.22	1.43 ± 48.64	0.647
HDL cholesterol (mg/dL)*	3.50 ± 16.99	−2.39 ± 8.47	0.124
LDL cholesterol (mg/dL)*	−3.50 ± 30.39	−3.56 ± 20.53	0.628
hs-CRP (mg/L)*	1.59 ± 6.25	−1.36 ± 6.50	0.251
White blood cell count (10^*∧*^9/L)	−0.13 ± 1.02	−0.42 ± 1.07	0.328
Hemoglobin (g/dL)*	−0.12 ± 0.51	0.73 ± 5.24	0.208
Hematocrit (%)*	−2.09 ± 7.30	−1.43 ± 1.11	0.258
Platelet count (10^*∧*^9/L)	−14.50 ± 23.21	−5.65 ± 29.32	0.226
Total protein (g/dL)*	−0.15 ± 1.31	0.00 ± 0.29	0.240
Albumin (g/dL)*	0.04 ± 0.14	0.02 ± 0.17	0.483
Total bilirubin (mg/dL)*	0.04 ± 0.14	0.00 ± 0.33	0.112
Direct bilirubin (mg/dL)*	0.01 ± 0.06	0.01 ± 0.12	0.568
SGOT (IU/L)*	−0.26 ± 6.20	−0.60 ± 3.47	0.971
SGPT (IU/L)*	−0.73 ± 10.41	−1.13 ± 5.40	0.869
*γ*GTP (IU/L)*	−1.26 ± 7.65	1.17 ± 12.64	0.487
Blood urea nitrogen (mg/dL)*	−1.43 ± 2.80	−1.52 ± 3.96	0.474
Serum creatinine (mg/dL)*	0.03 ± 0.10	0.00 ± 0.05	0.223
Uric acid (mg/dL)*	−0.09 ± 0.59	−0.10 ± 0.63	0.621
TSH (mIU/mL)*	0.13 ± 1.45	−0.14 ± 1.15	0.680
Free T4 (ng/dL)*	0.08 ± 0.35	0.07 ± 0.31	0.634

*t*-test; *P value using Mann-Whitney *U*-test; ^‡^ *P* < 0.05.

Values given are number of patients (%) or mean ± SD.

LDL: low-density lipoprotein; HDL: high-density lipoprotein; hs-CRP: highly sensitive C-reactive protein; SGOT: aspartate transaminase; SGPT: alanine transaminase; *γ*GTP: *γ*-glutamyltranspeptidase; TSH: thyroid stimulating hormone.

**Table 3:** Side effects during treatment of THI and placebo.

Side effects	THI	Placebo	*P* value
	(*n* = 30)	(*n* = 23)	
Anorexia grade 1*	0	1 (4.3)	0.434
Nausea grade 1	5 (16.7)	1 (4.3)	0.217
Indigestion grade 1	8 (26.7)	3 (13.0)	0.313
Hunger pain grade 1	1 (3.3)	0	1.0
Abdominal pain grade 1	1 (3.3)	0	1.0
Loose stool grade 1	2 (6.7)	3 (13.0)	0.642
Diarrhea grade 1	4 (13.3)	1 (4.3)	0.374
Constipation grade 1	2 (6.7)	1 (4.3)	1.0
Facial edema grade 1	1 (3.3)	0	1.0
Headache grade 1	1 (3.3)	0	1.0
Insomnia grade 1	2 (6.7)	1 (4.3)	1.0
Itching grade 1	2 (6.7)	0	0.499
Fatigue grade 1	1 (3.3)	1 (4.3)	1.0
Palpitation grade 1	0	1 (4.3)	0.434
Satiety grade 1	2 (6.7)	2 (8.7)	1.0
Relieve constipation grade 1	5 (16.7)	4 (17.4)	1.0
Good appetite grade 1	1 (3.3)	1 (4.3)	1.0
Increased gastric acid secretion grade 1	1 (3.3)	0	1.0
Suppression of appetite grade 1	3 (10.0)	4 (17.4)	0.451
Thirst grade 1	1 (3.3)	0	1.0

Chi-square test; *P value using Fisher's exact test.

Values given are number of patients (%).

Grading scale of Common Terminology Criteria for Adverse Events v3.0 (CTCAE).
